# Investigation into the Efficient Cooperative Planning Approach for Dual-Arm Picking Sequences of Dwarf, High-Density Safflowers

**DOI:** 10.3390/s25144459

**Published:** 2025-07-17

**Authors:** Zhenguo Zhang, Peng Xu, Binbin Xie, Yunze Wang, Ruimeng Shi, Junye Li, Wenjie Cao, Wenqiang Chu, Chao Zeng

**Affiliations:** 1 College of Mechanical and Electrical Engineering, Xinjiang Agricultural University, Urumqi 830052, China; xupeng9018@163.com (P.X.); 120240048@xjau.edu.cn (B.X.); wangyunze_531@163.com (Y.W.); 19199276782@163.com (R.S.); lijunyexjnydx@163.com (J.L.); 15265406750@163.com (W.C.); chuwenqiang2021@163.com (W.C.); chaozeng5054@163.com (C.Z.); 2 Key Laboratory of Xinjiang Intelligent Agricultural Equipment, Xinjiang Agricultural University, Urumqi 830052, China

**Keywords:** picking robot, dual-arm collaboration, ant colony optimization, path planning, picking trajectory

## Abstract

Path planning for picking safflowers is a key component in ensuring the efficient operation of robotic safflower-picking systems. However, existing single-arm picking devices have become a bottleneck due to their limited operating range, and a breakthrough in multi-arm cooperative picking is urgently needed. To address the issue of inadequate adaptability in current path planning strategies for dual-arm systems, this paper proposes a novel path planning method for dual-arm picking (LTSACO). The technique centers on a dynamic-weight heuristic strategy and achieves optimization through the following steps: first, the K-means clustering algorithm divides the target area; second, the heuristic mechanism of the Ant Colony Optimization (ACO) algorithm is improved by dynamically adjusting the weight factor of the state transition probability, thereby enhancing the diversity of path selection; third, a 2-OPT local search strategy eliminates path crossings through neighborhood search; finally, a cubic Bézier curve heuristically smooths and optimizes the picking trajectory, ensuring the continuity of the trajectory’s curvature. Experimental results show that the length of the parallelogram trajectory, after smoothing with the Bézier curve, is reduced by 20.52% compared to the gantry trajectory. In terms of average picking time, the LTSACO algorithm reduces the time by 2.00%, 2.60%, and 5.60% compared to DCACO, IACO, and the traditional ACO algorithm, respectively. In conclusion, the LTSACO algorithm demonstrates high efficiency and strong robustness, providing an effective optimization solution for multi-arm cooperative picking and significantly contributing to the advancement of multi-arm robotic picking systems.

## 1. Introduction

Safflower is a highly distinctive economic crop with medicinal, dyeing, oil-producing, and forage value [1,2]. Mature plants exhibit an umbrella-shaped structure with densely distributed capitula. Each safflower can be picked 3 to 5 times, but due to varying maturation times, selective and batch picking is required [3,4,5]. In complex picking environments, the umbrella-shaped distribution and pronounced height differences of safflower result in limited picking space. This constrains the operational range of single-arm picking devices, reducing the effective coverage per picking session and failing to meet practical operational demands. Therefore, it is crucial to develop multi-arm safflower-picking robots to enhance picking efficiency [6,7]. Currently, traditional path planning methods encounter challenges such as path crossing and low picking efficiency when applied to dual-arm safflower-picking scenarios, failing to meet the precise path planning requirements of such robots. Consequently, under conditions where safflower exhibits significant variation in maturation time and optimal picking occurs only during full bloom, it is imperative to optimize path planning to avoid the path crossing issues inherent in traditional methods. Overcoming the limitations of single-arm devices—restricted operational range and limited per-session coverage—is key to significantly improving efficiency of safflower picking, which remains a pressing problem to be addressed.

In recent years, multi-arm picking robots have become a research focus in the field of agricultural robotics, with numerous scientific achievements providing valuable insights for the study of dual-arm safflower picking robots [8]. Huang et al. [9] proposed a dual-arm apple harvesting robot system, which employs a multi-arm task planning method based on a genetic algorithm to optimize the target collection sequence for each arm, enhancing collaborative efficiency. Yin et al. [10] introduced a workspace task division method for a dual-arm mango-picking robot, incorporating a depth-first picking strategy. Compared to single-arm robots, the picking time was reduced by 48.38%. Marco Riboli et al. [11] defined the geometric path of the end-effector using a fifth-order B-spline curve, but this method is complex and computationally intensive. However, compared to single-arm safflower picking robots, multi-arm picking involves more complex task planning. In addition to considering the picking strategy of each robotic arm, additional attention must be given to task allocation between the arms [12]. Williams [13] used the two-dimensional Euclidean distance between fruits as a basis for clustering kiwifruits and assigning tasks to robotic arms. Xiong et al. [14] planned the collaborative picking tasks between robotic arms by setting a safety distance in the Cartesian coordinate system, but relying solely on the safety distance for task planning may not enable precise task allocation. In terms of path planning methods, Zhang et al. [15] introduced an elliptical repulsive potential field range and boundary potential field into the artificial potential field method, improved the potential field function, and added a distance factor to reduce the complexity of the environmental potential field. Cao et al. [16] employed an improved Rapidly-exploring Random Tree (RRT) algorithm to solve the path planning problem for lychee picking robots. Building on Cao et al.’s research, Liu et al. [17] designed a time-optimal Rapidly-exploring Random Tree (TO-RRT) algorithm for the path planning of citrus-picking robots, shortening the path distance. Fang et al. [18] proposed an enhanced RRT* algorithm (XN-RRT*) to address the issues of low path planning efficiency and suboptimal picking success rate in complex pitaya harvesting environments. Wu et al. [19] addressed the issue of traditional ant colony algorithms becoming trapped in local optima by adaptively adjusting the pheromone evaporation factor. However, the method generates paths with severe crossings, affecting path quality. Li et al. [20] used a cosine annealing strategy to enhance the expected heuristic factor and balance the global search ability of the algorithm. Kiani et al. [21] proposed an extended Grey Wolf Optimization (Ex-GWO) method to find an optimal, collision-free path between two points for the robot; however, the path exhibits significant randomness. In industrial multi-agent transportation, Chen et al. [22] achieved efficient task allocation and path planning through a marginal cost allocation heuristic strategy and a large neighborhood search metaheuristic improvement strategy, providing valuable insights for agricultural robot research in urban traffic flow prediction. Ali et al. [23] developed a multi-graph neural network model to capture dynamic spatiotemporal correlations, and their deep learning-based spatiotemporal modeling approach may offer new ideas for optimizing agricultural robot algorithms. Currently, many scholars have conducted in-depth research on task allocation and path planning, proposing various methods to optimize operational efficiency and quality. However, in the face of complex real-world environments, such as uneven crop distribution and robotic arm interference zones, existing algorithms struggle to balance the efficiency and accuracy of both task allocation and path planning.

Given the complexity of safflower growth characteristics and the harvesting environment, task allocation and path planning are crucial when the dual-arm collaborative safflower picking robot’s mechanical arms work in tandem. Based on the uneven distribution and significant height differences of safflowers, this paper conducts research on dual-arm collaborative path planning for picking. By designing reasonable picking paths and trajectories, the robot can collaborate efficiently, improving picking efficiency and reducing collision risks. Significant progress has been made in safflower harvesting research. Studies have explored safflower harvesting technology, with research on safflower identification and localization being relatively mature [24,25]. However, there are still technical bottlenecks in the field of harvesting path planning: the research on safflower harvesting path planning based on parallel robotic arms by Guo Hui et al. [26] is innovative, but the singularity problem faced by parallel robotic arms during operation may affect the continuity and stability of the harvesting path [27]. The research by Zhang et al. [28] on Cartesian coordinate system single-arm picking robots, while demonstrating the efficiency and accuracy advantages of this arm configuration, suggests that in the complex safflower planting environment—where plants are unevenly distributed, and height differences are prominent—there may still be room for improvement in terms of operational speed and overall efficiency. This warrants further exploration of multi-arm collaborative or adaptive adjustment solutions better suited for complex environments. The main contributions of this paper are as follows:

(1) The safflower dual-arm harvesting task is modeled as a Multiple Traveling Salesman Problem (MTSP). The K-means algorithm is used to cluster the harvesting points and divide the work areas. Combined with a load balancing strategy, tasks are assigned to the dual robotic arms, achieving cooperative path planning and zoning to reduce the risk of cross-area path conflicts.
(2) A dynamic weight heuristic strategy is introduced into the Ant Colony Optimization (ACO) algorithm. By integrating heuristic information from the inverse of local distances and the inverse of node global average distances, the state transition probabilities of path planning are optimized, allowing the algorithm to dynamically balance global search and local convergence during iterations.
(3) A 2-OPT local search strategy is employed to detect path crossing edges and reconstruct subpaths, eliminating redundant trajectory crossings, shortening the total length of the harvesting path, and improving the compactness and execution efficiency of path planning.
(4) A parallelogram harvesting trajectory optimized based on cubic Bezier curves is designed. By adjusting the control points, the trajectory curvature continuity is optimized, smoothing discrete path points into a continuous curve, thus enhancing the trajectory smoothness and motion stability of path planning.

The remainder of this paper is organized as follows: Section 2 analyzes the safflower picking scenario and introduces the robot’s operational principles. Section 3 presents the dual-arm planning method for the picking robot. Section 4 discusses the experimental results. Finally, Section 5 concludes the research and outlines future research directions.

## 2. Analysis of the Picking Scenario and Design of the Dual-Arm Picking Collaborative Robot

### 2.1. Analysis of the Characteristics and Challenges of Safflower Picking Scenarios

**Figure 1:**
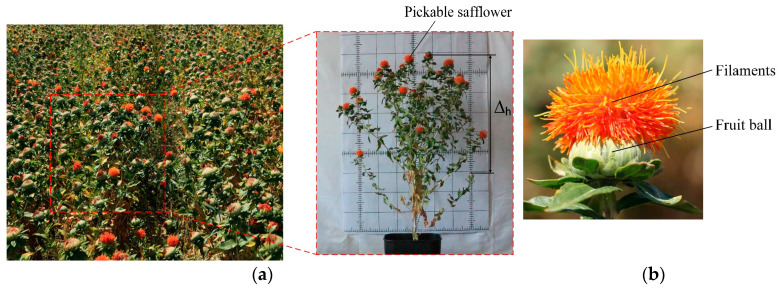
Safflower picking scenario. (**a**) Field environment of the safflower; (**b**) structure of the safflower.

The safflower picking environment presents several unique characteristics of both the environment and the crops. To achieve efficient mechanized picking and fully utilize the performance advantages of the picking equipment, the planning of picking paths and the design of equipment must overcome the following challenges in this complex working environment:

(1) During peak blooming, safflower distribution is random, so relying only on high-speed picking and capturing mechanisms reduces efficiency. Path planning for picking is essential.
(2) Safflower plant height varies between 400 and 950 mm, and fruit clusters are scattered with significant height differences, as shown in Figure 1a. Single-arm picking devices struggle with multi-target picking, leading to low efficiency.
(3) Safflower filaments are above the fruit clusters and scattered, requiring precise separation, as shown in Figure 1b. This scattered distribution complicates area division, causing path crossings and increasing the picking path length.

To address the picking challenges caused by environmental complexity, this study designs a dual-arm safflower picking robot based on the Cartesian coordinate system. By accurately describing the spatial position of the target safflower and planning the dual-arm collaborative motion path, efficient operations in complex environments are achieved, enhancing both safflower picking efficiency and quality.

### 2.2. Overall Plan for the Dual-Arm Safflower Picking Robot

**Figure 2:**
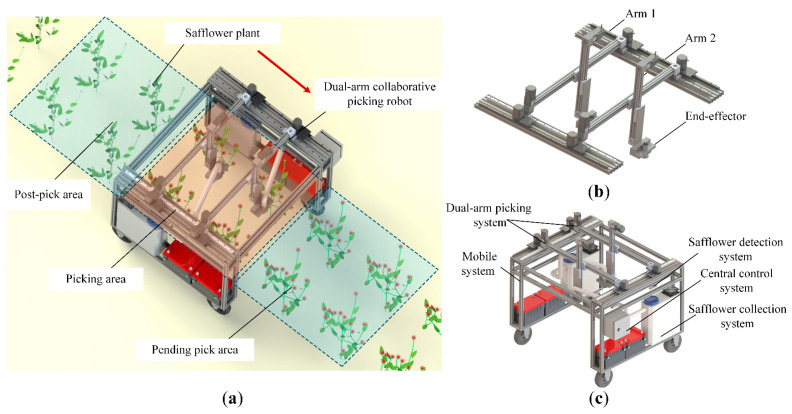
Safflower dual-arm collaborative picking robot. (**a**) Operational scenario diagram; (**b**) dual-arm picking system; (**c**) robot diagram.

The operational scenario of the dual-arm safflower-picking robot is illustrated in Figure 2a, with the core design focusing on the dual-arm system and path planning. The entire system centers on the dual-arm collaborative picking system, consisting of two safflower-picking robotic arms and end-effectors, as shown in Figure 2b. The dual-arm base is mounted on the upper part of a mobile platform, allowing for flexible, multidimensional movement, as shown in Figure 2c. During operation, the safflower detection system captures plant images and extracts the coordinates of the picking points, transmitting this information to the central control system. Based on the requirements of coordinated dual-arm operation, the system plans collision-free and high-efficiency motion paths, guiding the robotic arms and driving the end-effectors to follow the planned trajectories and perform filaments picking. The cut filaments are then collected by the picking system. Upon completing the current area, the robot moves to a new region, where the central control system replans the dual-arm motion paths using updated data, enabling continuous picking. This approach overcomes the limitations of single-arm devices and addresses the inefficiencies of traditional path planning, thereby achieving efficient safflower picking in complex environments.

### 2.3. Overall Framework for Dual-Arm High-Speed Path Planning Solution

**Figure 3:**
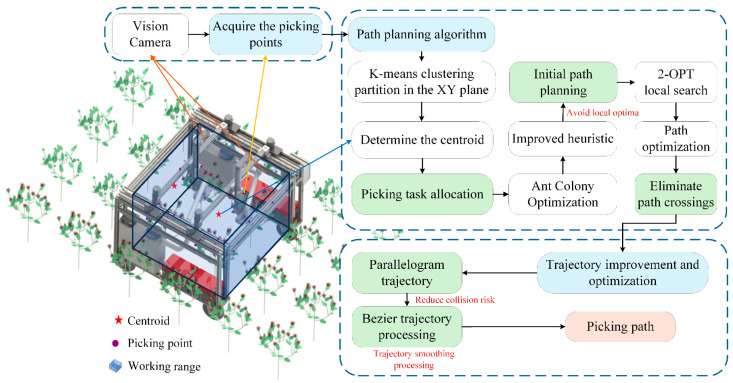
Overall framework of the high-speed path planning solution for the dual-arm safflower picking robot.

The overall framework for the high-speed path planning of the dual-arm safflower-picking robot is shown in Figure 3.

(1) A region division rule is established based on safflower height fluctuations and flower cluster distribution. The work area is split into harvestable and collision-prone zones using orthogonal planes. A load balancing strategy ensures no overlap between dual-arm operations, minimizing path length and laying the groundwork for path planning.
(2) The K-means algorithm clusters the discrete target points, projecting 3D points onto the x-y plane to create two sub-regions and their centroids. This enables task allocation between the robotic arms, reducing interference and providing input for the improved Ant Colony Optimization (ACO) algorithm.
(3) A dynamic weight heuristic is added to improve the ACO algorithm, addressing local optimality in disorganized target points. By integrating global path information and adjusting weight factors, the path selection balances pheromone concentration and global target importance, avoiding local optima.
(4) To resolve potential crossing redundancies in the preliminary paths, the 2-OPT strategy is used to optimize the path structure. By detecting crossing edges and removing/reconnecting subpaths, a better solution is generated, retaining the shortest distance options, and further enhancing the compactness and efficiency of the path.
(5) A parallelogram-shaped picking trajectory is designed to reduce path distance. To address vibration issues caused by sharp turns in the path, the Bezier curve is used to smooth and optimize the parallelogram trajectory, ensuring continuous acceleration during motion and creating an efficient and stable picking trajectory.

## 3. Picking Sequence Planning Method

### 3.1. Picking Area Division Method

**Figure 4:**
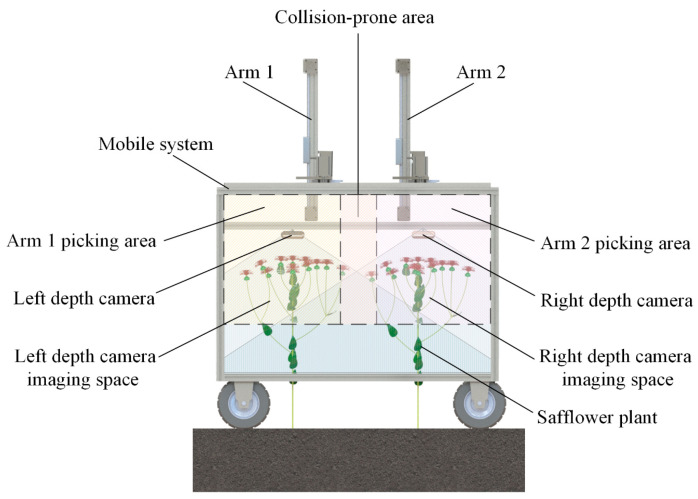
Safflower filament picking area diagram.

In safflower picking operations, the target safflowers exhibit varying heights and random distribution, making it difficult for the safflower dual-arm collaborative picking robot to locate targets quickly. This often results in repeated or missed picks, and the robotic arms may interfere with each other, reducing picking efficiency. Therefore, area division is essential to organize the randomly distributed safflowers, thereby improving picking efficiency. To address this, this section outlines the safflower filament picking area division method, as shown in Figure 4. Based on the growth characteristics of the safflower plants and the spatial relationship between the depth camera’s imaging area and the robotic arm’s working space, the picking area is divided into pickable and collision-prone zones using an orthogonal coordinate plane. For the collision-prone zones, a task load-balancing-based priority allocation strategy is adopted, assigning these areas to the robotic arm with the shortest picking path and the least task load, thereby preventing interference between the two arms.

Furthermore, the dual-arm collaborative picking path planning problem is formulated as an MTSP, where the safflower picking end-effectors are regarded as “salesmen” and the picking points as “cities”. The MTSP model is used to plan the optimal picking path, ensuring that the total movement distance and picking time of the end-effectors are minimized. In the MTSP formulation for dual-arm collaborative safflower picking, each task point is defined as the 3D coordinate of a safflower picking target.

A group of task points:

(1) $$C=\left\{\left.1,2,\ldots ,n\right\}\right.$$

where *C* is the set of safflower harvesting task points, and 1, 2, …, *n* represent the *n* task points.

The decision variable is denoted by $x_{ij}^{k}$ with values as shown in Equation (2).

(2) $$x_{ij}^{k}=\left\{\begin{array}{cc} 1, & When\;robotic\;arm\;k\;moves\;from\;task\;po\mathrm{int}\;i\;to\;task\;po\mathrm{int}\;j\\ 0, & Otherwise \end{array}\right.$$

The distances between task points are calculated using the Euclidean distance formula:

(3) $$d_{ij}=\sqrt{{\left(x_{i}-x_{j}\right)}^{2}+{\left(y_{i}-y_{j}\right)}^{2}+{\left(z_{i}-z_{j}\right)}^{2}}$$

where $d_{ij}$ is the Euclidean distance between the *i*-th and *j*-th task points, $x_{i}$ and $x_{j}$ is the x-axis coordinate of the *i*-th and *j*-th task points, $y_{i}$ and $y_{j}$ is the y-axis coordinate of the *i*-th and *j*-th task points, and $z_{i}$ and $z_{j}$ is the z-axis coordinate of the *i*-th and *j*-th task points.

Constraint: Each safflower harvesting task point must be visited once.

Objective 1: Minimize the total travel distance of the two robotic arms, as shown in Equation (4).

(4) $$min\;Z=\sum_{k=1}^{2}\sum_{i=1}^{n}\sum_{j=1}^{n}d_{ij}x_{ij}^{k}$$

where *min Z* is the minimum total travel distance of the robotic arms, *n* is the number of task points. By solving the MTSP model, the optimal harvesting path for the dual-arm safflower harvesting end-effectors can be obtained through this process.

Objective 2: Minimize the harvesting time, as shown in Equation (5).

(5) $$min\;T=\max\left\{\frac{\sum_{i,j\in V_{k}}^{n}d_{ij}x_{ij}^{k}}{v}+n_{k}t_{p}\right\}$$

where minT is the minimum picking time, $V_{k}$ is the set of task points assigned to robotic arm *k*, *v* is the velocity of the end-effectors of the robotic arms, $n_{k}$ is the number of task points assigned to robotic arm k (i.e., $n_{k}=\left|V_{k}\right|$), $t_{p}$ is the time for a single picking operation.

The dual-arm path planning problem is formulated as the MTSP and addressed through LTSACO, aiming to determine the optimal picking path for the end-effectors in the safflower picking task.

### 3.2. Safflower Picking Planning Method

**Figure 5:**
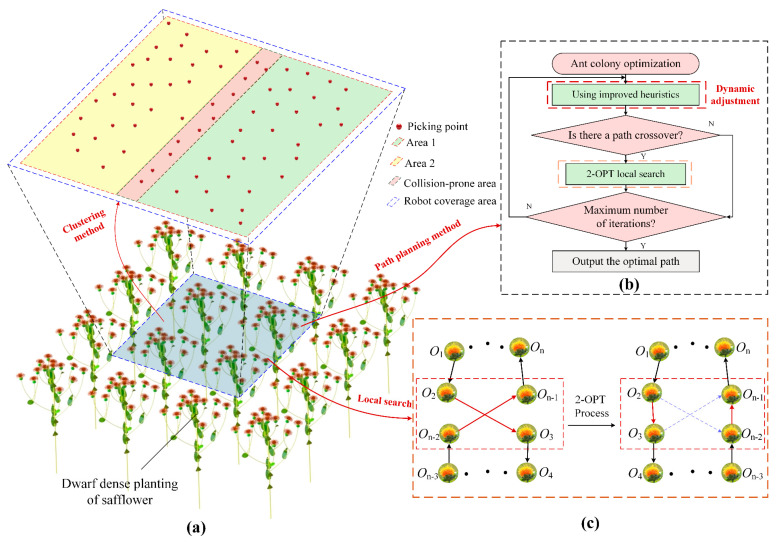
Picking sequence planning scheme. (**a**) K-means clustering process; (**b**) path planning method; (**c**) 2-OPT optimization process.

In complex environments, the dual-arm collaborative safflower picking robot must optimize the picking path to balance the shortest path and safety; the picking sequence planning scheme is illustrated in Figure 5. First, the K-means clustering algorithm is used to divide the picking area into multiple sub-regions, achieving reasonable task allocation and mitigating the impact of scattered target points. The improved ant colony optimization algorithm is then applied to further optimize the path, addressing the issue of long paths and local optimum. To further enhance the path quality, the 2-OPT local search strategy is used to eliminate path crossings, shorten the total travel distance, and improve the trajectory quality.

#### 3.2.1. Picking Clustering Method

The rational regional division is key to efficient path planning for the safflower dual-arm collaborative picking robot. In collaborative dual-arm operations, directly assigning work areas based on tool coverage can lead to overlapping operational zones, increasing the risk of robotic arm collisions. To address this, the K-means clustering algorithm is used to divide the picking area into two regions (i.e., *k* = 2), as shown in Figure 5a. Considering the characteristics of the dual-arm collaborative system, the covered picking area is partitioned into two sub-regions. The algorithm quickly clusters picking points according to their spatial distribution, effectively handling the complex and dense distribution of safflower clusters. Clustering is constrained to the x–y plane to avoid computational complexity caused by height differences in three-dimensional space, thereby reducing regional interference and the risk of arm collisions during operation and laying the foundation for subsequent path planning.

#### 3.2.2. Improved ACO Algorithm

Known as a classic path planning algorithm, the ant colony optimization mimics the foraging behavior of ants and utilizes the pheromone release mechanism to identify the shortest path [29]. In the ant colony optimization, ants select paths based on pheromone concentration, and they also release pheromones, creating a positive feedback mechanism. This allows the ant colony to find the relatively shortest path between the nest and food, even when obstacles are encountered during foraging. After completing the safflower picking area division, the traditional ant colony optimization often gets stuck in local optima when handling the chaotic distribution of safflowers in sub-areas for picking path planning. To optimize the path planning process, this section employs an improved ant colony optimization to optimize the picking order in each picking area.

In the ACO algorithm, the state transition probability during the foraging process primarily depends on the pheromone concentration and heuristic information along the path. Assume that in a static and known 3D environment, at time *t*, ant *k* (*k = 1, 2, …, M*) located at task point *i* needs to select a path to move to position *j*. The process is as follows: first, the ant calculates the transition probability $P_{ij}^{k}\left(t\right)$ for all possible paths surrounding position *i* using Equation (6). Subsequently, the target position *j* for the next move is determined using the roulette wheel selection rule.

(6) $$P_{ij}^{k}(t)=\left\{\begin{array}{cc} \frac{{\left[\tau_{ij}(t)\right]}^{\alpha }\cdot {\left[\eta_{ij}\right]}^{\beta }}{\sum_{l\in allowed_{k}}{\left[\tau_{ij}(t)\right]}^{\alpha }\cdot {\left[\eta_{ij}\right]}^{\beta }} & j\in allowed_{k}\\ 0, & otherwise \end{array}\right.$$

(7) $$\eta_{ij}(t)=\frac{1}{d_{ij}}$$

where $\tau_{ij}\left(t\right)$ is the pheromone concentration along the path at iteration t, with *t* representing the current iteration number. α is the pheromone concentration factor, *β* is the heuristic information factor. $allowed_{k}$ is the set of candidate cities, $l\in allowed_{k}$ is the target point *l* that has not yet been visited by the previous ant, *k*, $\eta_{ij}$ is the heuristic information from position *i* to position *j*, $d_{ij}$ is the Euclidean distance from point *i* to point *j*.

The ACO algorithm demonstrates strong global search capabilities in the single Traveling Salesman Problem (TSP). However, in the Multiple Traveling Salesman Problem (MTSP) involved in the safflower dual-arm collaborative picking robot, the simultaneous participation of multiple agents often leads to excessive pheromone concentration, resulting in insufficient solution diversity and convergence to local optima [30]. To address this issue, this study proposes an indirect expectation heuristic strategy combined with dynamically regulated weights based on the ACO algorithm. This method integrates global path information to prevent pheromone over-concentration and enhance both the diversity of path selection and the global search capability in safflower picking. By incorporating global path structure information, the influence of local information on ant decision-making is preserved, preventing all ants from converging on the same picking path and reducing the excessive interference of pheromones in the path selection process.

In the ACO algorithm, the heuristic information $\eta_{ij}\left(t\right)$ typically considers only the direct distance between the current node and the target node, neglecting the global path structure. This can limit path selection to local areas and reduce the global search capability. The improved indirect expectation heuristic strategy combined with dynamically regulated weights is shown in Equation (8). The improved heuristic information from node *i* to node *j* is defined as follows:

(8) $$\eta_{ij}=\omega_{1}\cdot \frac{1}{d_{ij}}+\omega_{2}\cdot \frac{\sum_{k\in V\backslash \{i,j\}}\frac{1}{d_{jk}}}{\left|V\right|-2}$$

where $d_{ij}$ is the Euclidean distance from node *i* to node *j*, $\sum_{k\in V\backslash \{i,j\}}\frac{1}{d_{jk}}$ is the reciprocal of the average distance from a node to all other nodes, $\left|V\right|$ is the total number of nodes, $\omega_{1}$, $\omega_{2}$ is the dynamic weight factor, satisfying the condition. The dynamic weight factor is dynamically adjusted through the following formula:

(9) $$\omega_{1}=\frac{1}{1+e^{-\lambda \cdot (t-t_{0})}}$$

(10) $$\omega_{2}=1-\omega_{1}$$

where λ is the control factor, t is the current iteration number, $t_{0}$ is the intermediate point in the iteration, which controls the timing of the weight switching.

#### 3.2.3. 2-OPT Local Search

In the task of coordinated picking of safflowers by dual robotic arms, preliminary path planning using a dynamic heuristic ant colony optimization may result in path crossings, leading to interference or even collisions between the robotic arms. Additionally, this increases the total length of the picking path, thereby reducing efficiency. Therefore, optimizing the path and eliminating crossings are crucial for enhancing both operational efficiency and safety. The 2-OPT local search, as a classical combinatorial optimization algorithm, can effectively address the path crossing issue and has demonstrated excellent performance in path planning [31]. To this end, the 2-OPT local search strategy is employed to improve the ant colony optimization. The process of the local search strategy is shown in Figure 5c. In the illustration on the left, the target points $O_{1}$ to $O_{n}$ are sequentially connected, forming an initial path where $O_{2}$ is connected to $O_{3}$ and $O_{n-2}$ is connected to $O_{n-1}$, resulting in path crossings. After 2-OPT optimization, the new path is displayed in the illustration on the right. By inspecting the crossing sections of the path, crossing edges are removed, and the subpaths are reconnected to generate a better solution. The improvement is accepted only if the new path length is shorter than the original one, effectively optimizing the path structure and reducing the total safflower picking path length.

### 3.3. Improvement and Smoothing Optimization of the Picking Trajectory

After completing the path planning and local search, further refinement of the picking motion path is necessary to enhance both the efficiency and safety of the robotic arms during the actual safflower picking process. A well-designed motion path, considering the growth characteristics of the safflower plants and the spatial distribution of the target points, can prevent collisions between the robotic arms and the branches while optimizing the continuity and stability of the picking actions. Therefore, this section presents a design based on cubic Bézier curve optimization for the parallelogram trajectory of the picking path. By optimizing the parallelogram trajectory, a more reasonable safflower picking motion trajectory is generated, providing an appropriate trajectory for the efficient operation of the dual-arm collaborative safflower picking robot.

#### 3.3.1. Improvement of the Parallelogram Trajectory

**Figure 6:**
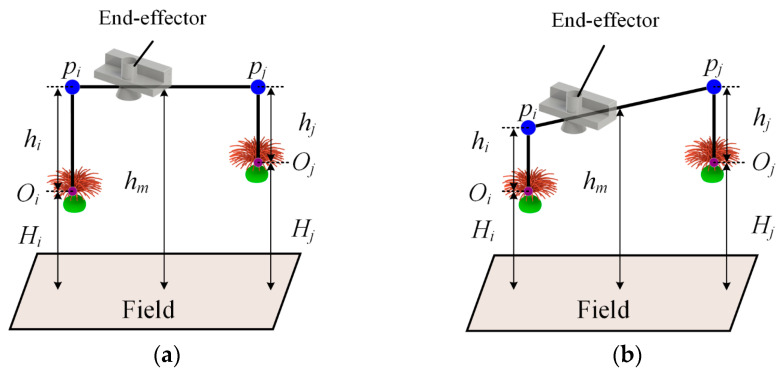
Improved trajectory diagram. (**a**) Gantry path; (**b**) improved parallelogram path.

**Figure 7:**
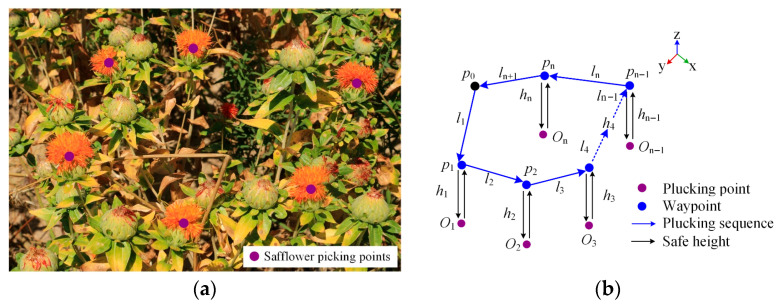
Safflower picking trajectory diagram. (**a**) Safflower plant; (**b**) safflower picking path diagram.

In safflower picking operations, multiple flower heads are commonly densely distributed on individual plants, with significant height variations. Traditional end-effectors follow a gate-type trajectory, as shown in Figure 6a. The two picking points, $O_{i}$ and $O_{j}$, correspond to heights $H_{i}$ and $H_{j}$, with differing safe heights $h_{i}$ and $h_{j}$. During point-to-point picking, the safety clearance height at different picking points is unequal. Although this avoids collision with branches and flower heads, the total path length increases in multi-target operations, limiting picking efficiency. To address this, an improved parallelogram trajectory, as shown in Figure 6b, is proposed. By unifying the dropping height of adjacent picking points, the movement distance of the end-effector is minimized, achieving path optimization. This trajectory focuses on height control, and as the dual-arm picking system moves along the planned trajectory, it minimizes the height variation between picking points to avoid path deviations. In addition, considering the dense distribution of flower heads in safflower fields, as shown in Figure 7a, the middle height of the path, hm, is further optimized, as shown in Figure 7b, thereby preventing collisions with plant branches and flower heads from a spatial perspective. This approach achieves both obstacle avoidance safety and the shortest path collaboration.

#### 3.3.2. Cubic Bézier Curve Trajectory Optimization

In the safflower picking operation, sharp turns in the picking path can lead to abrupt changes in robotic arm acceleration and intensified vibrations, ultimately reducing picking precision. Therefore, path optimization is essential. Bézier curves, known for their smoothness and shape controllability, are effective in connecting path points smoothly and adapting to the irregular distribution of safflowers. The choice of curve order significantly impacts the effectiveness of path optimization. Given the sparse and irregular spatial distribution of safflowers in the field, picking path planning must prioritize operational efficiency. Although higher-order Bézier curves offer stronger fitting capabilities, they are computationally intensive and time-consuming to generate. In contrast, third-order Bézier curves meet basic shape requirements while maintaining relatively low computational complexity, enabling rapid path generation. Furthermore, to ensure smooth and precise robotic arm movement, the trajectory must exhibit continuous curvature. A cubic Bézier curve, when designed with properly positioned control points, can satisfy second-derivative continuity, thereby reducing the impact of sudden curvature changes [32]. The expression for the cubic Bézier curve is as follows:

(11) $$B(t)={\left(1-t\right)}^{3}P_{0}+3t{\left(1-t\right)}^{2}P_{1}+3t^{2}(1-t)P_{2}+3t^{3}P_{3},t\in [0,1]$$

where $B\left(t\right)$ is the parametric equation of a Bézier curve, $P_{0}$ and $P_{3}$ are adjacent control points, $P_{1}$ and $P_{2}$ are the intermediate control points of the cubic Bézier curve.

**Figure 8:**
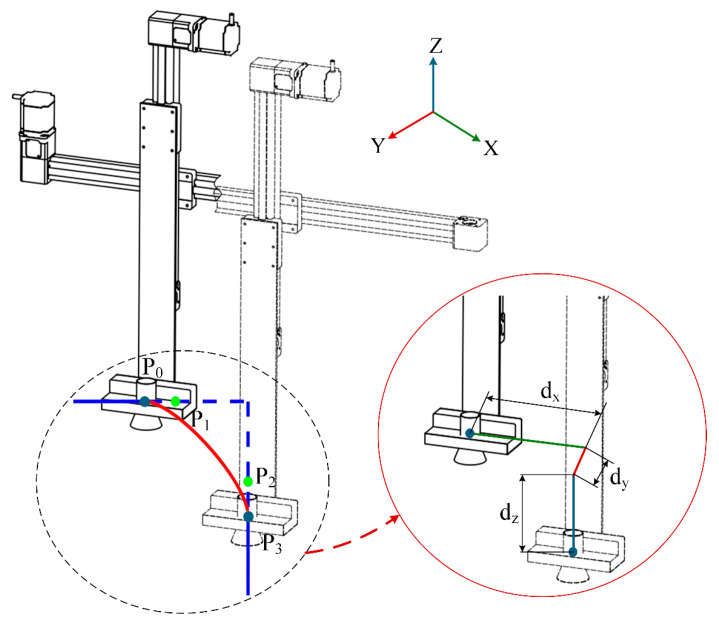
Cubic Bézier curve diagram. Note: *P*_0_, *P*_1_, *P*_2_, and *P*_3_ are the control points of the cubic Bézier curve. dx, dy, and dz represent the motion quantities in the x, y and z directions before and after the optimization of the Bézier curve.

The cubic Bézier curve is shown in Figure 8. The first control point, $P_{0}$, represents the starting point of the curve, while the final control point, $P_{3}$, marks the endpoint. The intermediate control points, $P_{1}$ and $P_{2}$, lie outside the curve, and their direction and distance determine the starting tangent direction, ensuring that the curve connects smoothly to the two straight lines. By adjusting the number and positions of the control points, various forms of Bézier curves can be flexibly constructed. In path planning, after smoothing the Bézier curve, a trajectory closer to the theoretical driving path can be generated, enabling smoother motion control. The smoothing process involves the following steps: first, extracting key turning points from the planned picking path; then, using the cubic Bézier curve to interpolate the path segments between adjacent turning points, thereby enhancing the smoothness of the path.

### 3.4. Running Process of the Safflower Picking Planning Algorithm

To clearly illustrate the logical relationships and execution sequence of each module, a detailed pseudo-code of the algorithm is shown below (Algorithm 1):
**Algorithm 1**. The LTSACO **Input:** 3D-points, *m*, *ρ*, *α*, *β*, *k*, *G* **Output:** Best path length, Run time1 Time start ← Run time start count2 $K-cluster=\left(X_{distance},Y_{distance}\right)$ ← K-means cluster points3 $d_{ij}=\left(X_{distance},Y_{distance},Z_{distance}\right)$ ← Euclidean distance4 $D=\{d_{ij}|i,j=\left(1,2,\ldots ,n\right)\}$ ←$d_{ij}$ consists distance matrix *D*5 **for** each *i* in [1,*k*] **do**6  Initialize heuristic matrix τ
7  **for** each t=1 to *G* **do**8    Compute dynamic weight9    $\omega_{1}=\frac{1}{1-e^{-\lambda \left(t-t_{0}\right)}}$, $\omega_{2}=1-\omega_{1}$, $t_{0}=\frac{t}{2}$
10   Compute $\eta_{ij}$
11   $\eta_{ij}=\omega_{1}\cdot \frac{1}{d_{ij}}+\omega_{2}\cdot \frac{\sum_{k\in V\{i,j]}\frac{1}{d_{jk}}}{\left|V\right|-2}$
12   **for** each ant **do**13      Using improved rules to improve calculations14      $P_{ij}^{k}\left(t\right)=\left\{\begin{array}{c} \frac{{\left[\tau_{ij}\left(t\right)\right]}^{\alpha }\cdot {\left[\eta_{ij}\right]}^{\beta }}{\sum_{l\epsilon allowe\;\lambda_{k}}{\left[\tau_{ij}\left(t\right)\right]}^{\alpha }\cdot {\left[\eta_{ij}\right]}^{\beta }}\\ 0 \end{array}\right.$
15    **end**16    Update best path, heuristic τ
17  **end**18  2-OPT local search ← Remove crossing edges19 **end**20 Cubic Bézier cuve trajectory optimization21 Time end ← Run time stop count22 return Best path length, Run time

The specific steps for algorithmically searching the optimal safflower picking path are as follows:

(1) The key parameters in the initialization algorithm are shown in Table 1.
(2) Obtain the 3D coordinate data of the safflower picking points by image processing techniques, then construct a complete 3D model of the picking points based on this data to provide accurate target point information for path planning.
(3) Apply the K-means clustering algorithm to divide the picking area into several sub-tasks based on the 3D Euclidean distance between picking points, with each sub-task containing multiple target picking points.
(4) Optimize the decision-making process for selecting the safflower picking path by adjusting the heuristic factors, enabling the algorithm to more efficiently converge toward the optimal solution while reducing excessive pheromone concentration.
(5) Use the 2-OPT local search strategy to optimize the path. By swapping two edges in the safflower picking path, this step eliminates path intersections and redundancies, shortens the total path length, and improves operational efficiency.
(6) After reaching the maximum number of iterations *G*, output the optimized safflower picking path plan and record the optimal path information.

## 4. Results and Discussion

The experiments were conducted on a Windows 11 64-bit computer equipped with an NVIDIA GeForce GTX 1660 Ti GPU (Nvidia, Santa Clara, CA, USA), an Intel i7-9750H CPU@ 2.6 GHz (Intel, Santa Clara, CA, USA), and 16 GB of RAM. MATLAB 2023a was used as the platform for running the code.

### 4.1. Selection of Optimal Configuration Parameters

The parameter settings of the ACO algorithm have a significant impact on both the algorithm’s runtime and the quality of the solution. However, there is currently no well-established parameter tuning method available in existing research. Therefore, it is necessary to determine the initial parameter settings of the ACO algorithm through experimental analysis [33,34]. To enhance the adaptability of the improved algorithm in scenarios with densely distributed safflowers, parameter tuning experiments were conducted in a simulated safflower-picking environment with dimensions of 1200 × 1200 × 1000 mm. A single-variable control approach was employed to compare the algorithm’s performance under different parameter settings, and the average results of 10 independent runs were selected as the experimental data for determining the optimal parameter combination. Key parameters that influence the performance of the ACO algorithm include the ant population (*m*), the pheromone factor (*α*), the heuristic function factor (*β*), and the pheromone volatilization factor (*ρ*). These parameters jointly influence the algorithm’s performance and need to be adjusted through experiments to identify the optimal configuration.

**Figure 9:**
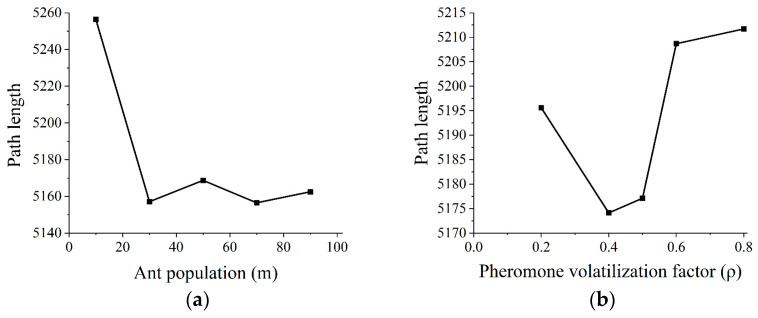
The trend of planning results under different parameters. (**a**) Trend of planning results with varying ant population; (**b**) trend of planning results with varying pheromone volatilization factor.

In the experiment on parameter selection for the ant colony optimization in safflower dual-arm path planning, the primary objectives are to minimize path length, maximize picking efficiency, and reduce collisions. The values for each parameter are as follows: *m* = {10, 30, 50, 70, 90}, *α* = {1, 2, 3, 4, 5}, *β* = {1, 3, 5, 7, 9}, and *ρ* = {0.2, 0.4, 0.5, 0.6, 0.8}, with default values of *m* = 20, *α* = 1, *β* = 5, and *ρ* = 0.5. In the experiment, a univariate control method is used, fixing the other two parameters while adjusting each parameter one at a time and recording the path length of the safflower picking. Each parameter combination is tested in 10 independent trials, with the best average result taken from the 10 trials to reduce randomness. The evaluation results of different parameter combinations are compared to select the optimal parameter set for improving path planning performance. Under default parameters, the impact of ant population (*m*) and pheromone volatilization factor (*ρ*) on the planning results is simulated using training group data, as shown in Figure 9.

The experimental results indicate that as the number of ants increases, the overall shortest path for safflower picking tends to decrease. The minimum average shortest path length occurs when the number of ants is 30. When the number of ants exceeds 30, the path length slightly decreases further, but as the number of ants increases, the computational workload and the required planning time also increase, which hinders the ability to obtain results quickly. Based on the studies by Sun et al. [35] and Wang et al. [36], it can be understood that when the number of ants is equal to the number of safflowers picking points to be planned, the path planned to use the improved ant colony optimization is more optimal. Therefore, the actual number of safflowers picking points within the planning area should be used as the number of ants in the ant colony optimization for path planning. The optimal pheromone volatilization factor (ρ) is 0.4.

**Figure 10:**
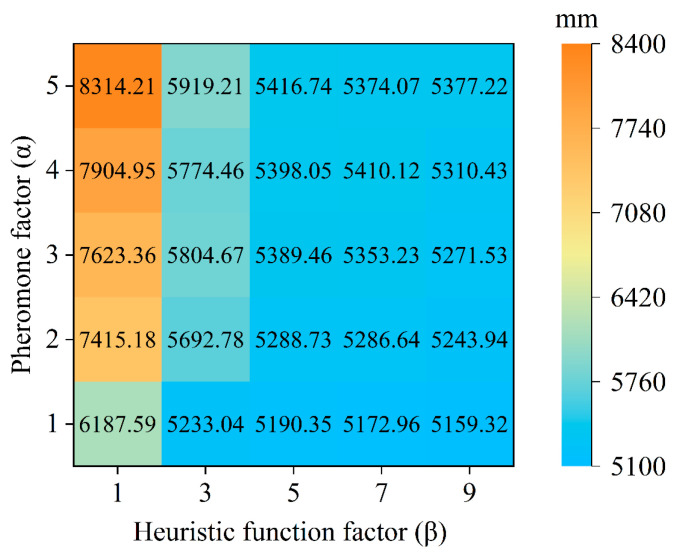
Heatmap of the impact of pheromone factor (*α*) and heuristic function factor (*β*) combination parameters on safflower picking path distance.

To determine the evaporation factor in Equation (6), *α* is set to {1, 2, 3, 4, 5} and *β* to {1, 3, 5, 7, 9}, with the remaining parameters set to their default values. The experimental results are shown in Figure 10.

The pheromone factor *α* and the heuristic function factor *β* significantly affect the path distance. When *β* is fixed, an increase in *α* leads to a reduction in the overall path distance. For example, when *β* = 5, as *α* decreases from 5 to 1, the path distance decreases from 5416.74 mm to 5190.35 mm, indicating that lowering *α* can optimize the path. When *α* is fixed, an increase in *β* results in a decrease in the path distance. For instance, when *α* = 5, as *β* increases from 1 to 9, the path distance decreases from 8314.21 mm to 5377.22 mm, suggesting that a larger *β* leads to a shorter path. Among the parameter combinations, the one with *α* = 1 and *β* = 9 yields the shortest average path distance of 5159.32 mm. Although other configurations perform relatively well, most of their path distances exceed the optimal value. Therefore, *α* = 1 and *β* = 9 represent the optimal parameter combination.

The initialization parameters for the code are shown in Table 2.

### 4.2. Module Ablation Test

To verify the effectiveness of the LTSACO path planning algorithm in the actual safflower dual-arm collaborative picking robot system, a collaborative task planning experiment was conducted using the robot’s hardware and software platform. According to the preliminary field survey of the safflower patch, the number of safflowers available for picking within the robot’s operational range is between 20 and 73, with an average of approximately 47 flowers. To demonstrate the effectiveness of the K-means clustering algorithm, the improved heuristic method, and the 2-OPT local search strategy in reducing path length in the improved ant colony optimization, an ablation experiment was designed. This experiment aims to verify the effectiveness of the improvements and each module in path optimization. All versions used the initial parameters from Section 4.1 and the same termination conditions, with 47 safflower picking points. Each scenario was run 10 times, and the average trial data was recorded to ensure fairness in the experiments. The results are shown in Table 3.

This section systematically analyzes the impact of the K-means, dynamic heuristic, and 2-OPT modules on path planning performance. The results show that when only the K-means module is used, dividing the tasks into two operational regions and assigning them to two robotic arms reduces the path length from 13,115.56 mm to 11,867.66 mm, a 9.51% decrease. However, the runtime increases from 0.5416 s to 1.2535 s. With the addition of the dynamic heuristic module, the path length further decreases to 11,818.73 mm, while the runtime slightly rises to 1.2659 s. When all modules are enabled, the path length reaches an optimal value of 11,780.10 mm, representing a 10.18% reduction compared to the baseline model, and the runtime is 1.2132 s. These experimental results indicate that, due to the complexity of the safflower picking path, the complete algorithm framework leads to an increase in runtime. Nevertheless, the collaborative effect of the modules significantly optimizes the path length, demonstrating the superiority of the approach in terms of path planning performance.

To further illustrate the advantages of dual-arm coordinated picking over single-arm picking, this section employs the K-means algorithm to assign picking tasks to two robotic arms designed for safflower picking. A traversal scheme is used to determine the picking order. For comparison, a single-arm robotic picking system is used to pick 47 target safflowers as a control. In the experiment, the time to pick one safflower with a single arm is set to 3 s, and the end-effector’s travel speed is set to 0.1 m/s. The experimental results are shown in Table 4.

The results indicate that the total picking time for the single-arm operation is approximately 141 s, with the traversal path planning accounting for around 117.79 s, resulting in a combined duration of about 258.79 s. The total time required for the single-arm picking scheme is approximately 1.69 times that of the dual-arm scheme. If the safflowers are evenly distributed between the two picking areas, allowing for balanced task allocation, the picking time could be further reduced. The significant advantage of dual-arm picking is primarily attributed to task allocation, where the K-means algorithm assigns picking tasks to two regions based on their spatial distribution. During dual-arm coordinated picking, each robotic arm operates within its designated region, minimizing long-distance travel and reducing the time required for path traversal. Moreover, the ability of both arms to operate simultaneously further enhances efficiency.

### 4.3. Trajectory Comparison Analysis

**Figure 11:**
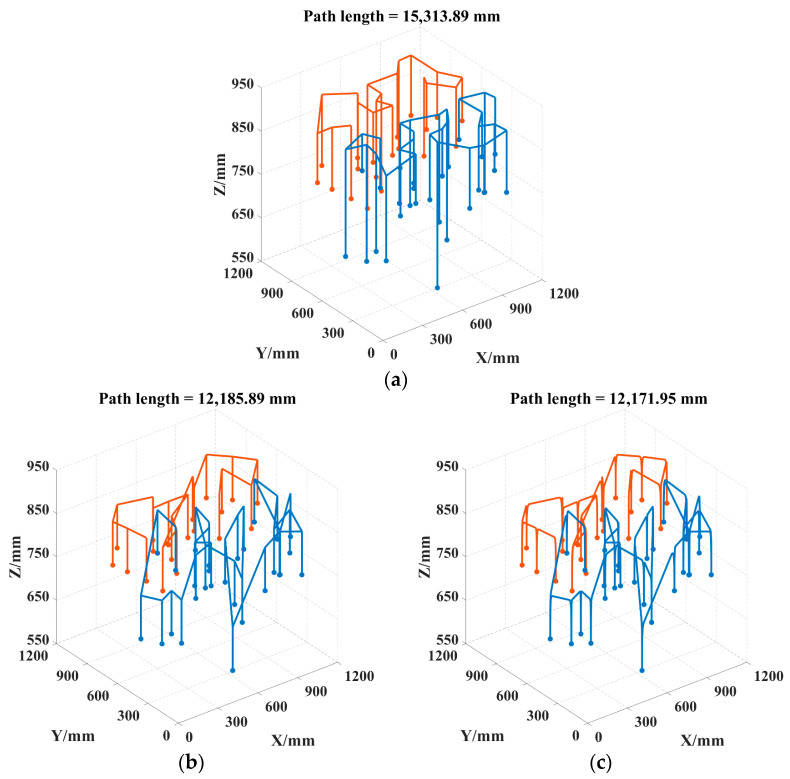
Trajectory comparison diagram. (**a**) Gantry trajectory; (**b**) parallelogram trajectory; (**c**) cubic Bézier curve-improved trajectory. Note: Different colors represent the motion trajectories of various picking robotic arms.

To further analyze the advantages of the improved trajectory in practical safflower picking applications, this section compares the path lengths of three picking trajectories: the gate-shaped trajectory, the quadrilateral trajectory, and the improved trajectory. The experiment involves path planning for 47 picking points using the MATLAB 2023a simulation platform. For each trajectory, the total path length is recorded. The resulting paths are shown in Figure 11.

The experimental results show that, in the test with 47 safflower picking points, the path distance for the gate-shaped trajectory was 15,313.89 mm, the unmodified parallelogram trajectory was 12,185.89 mm, and the parallelogram trajectory after improvement with the cubic Bézier curve was 12,171.95 mm. The results indicate that, compared to the traditional gate-shaped trajectory and the standard parallelogram planning method, the improved trajectory demonstrates better optimization in both path length and corner positioning.

In complex paths, the Bézier curve can replace multiple straight-line segments with a single continuous curve by adjusting the direction of the control points, shortening the total path. By setting control points, the curvature continuity of the trajectory is achieved, avoiding the redundancy of paths caused by traditional straight-line segment stitching. The parallelogram trajectory requires frequent turns due to geometric constraints. At the same time, the Bézier curve reduces the number of discrete turning points through its smooth transition characteristics, thus minimizing mechanical vibrations caused by sharp turns and further optimizing overall operational efficiency. The experimental data shows that the parallelogram trajectory reduced the path by 20.43% compared to the gate-shaped trajectory, and the Bézier curve-improved trajectory reduced the path by 0.11% compared to the parallelogram trajectory, and 20.52% compared to the gate-shaped trajectory. These experimental results validate the effectiveness of the Bézier curve in optimizing the safflower dual-arm picking path.

### 4.4. Comparison Analysis of Similar Algorithms

Based on the survey data from Section 4.2, experiments were conducted by selecting 20, 47 (the average), and 73 safflower picking points in three different scenarios. The LTSACO, DCACO, IACO, and ACO algorithms were used for task planning simulation tests. To minimize the impact of partitioning, each algorithm incorporated the K-means partitioning algorithm for comparison. Table 5 presents the results of the four algorithms for different numbers of safflower picking points.

As shown in Table 5, the picking path lengths for the ACO, DCACO, IACO, and LTSACO algorithms were recorded for 20, 47, and 73 safflower picking points. Each algorithm was independently run 10 times for each number of points. The best and worst path values, as well as the algorithm runtimes, were recorded for each run, and the mean and standard deviation of the path lengths were calculated.

**Figure 12:**
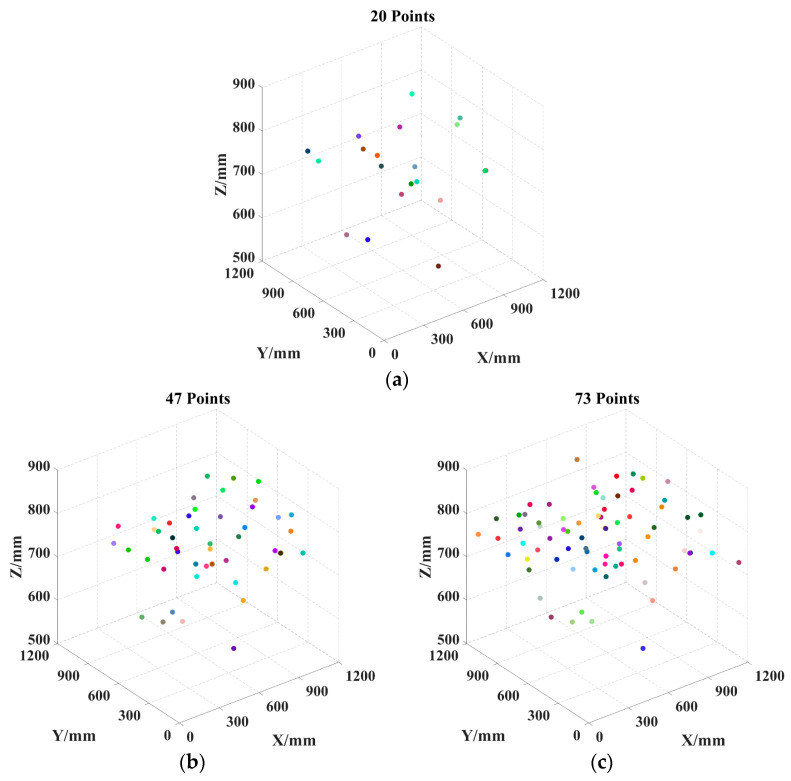
Scatter plots for different numbers of picking points. (**a**) Scatter plot for 20 picking points; (**b**) scatter plot for 47 picking points; (**c**) scatter plot for 73 picking points. Note: Each dot represents a safflower picking point.

**Figure 13:**
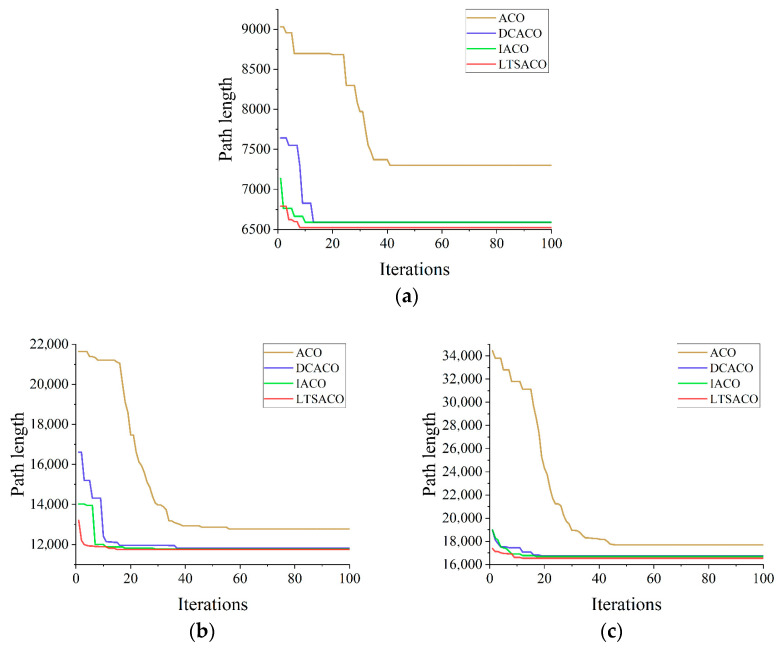
Iteration diagrams of four algorithms for different picking points. (**a**) Iteration diagram for 20 picking points; (**b**) iteration diagram for 47 picking points; (**c**) iteration diagram for 73 picking points.

To better visualize the picking path, the 3D coordinates of the picking points for safflower picking are presented, as shown in Figure 12. As can be seen in Figure 13, the convergence speed of DCACO and IACO is superior to that of ACO for different numbers of safflower picking points. According to Table 5, the LTSACO algorithm demonstrates significant advantages in path length optimization compared to ACO, DCACO, and IACO across different picking point scenarios. In the 20, 47, and 73 target point scenarios, the average path lengths of LTSACO were reduced by 11.06%, 9.33%, and 7.51%, respectively, compared to ACO, with an average reduction of 9.30%. Compared to DCACO, LTSACO reduced the path length by 3.29%, 0.96%, and 1.10%, with an average reduction of 1.78%. Compared to IACO, the reductions were 1.17%, 0.61%, and 0.56%, with an average reduction of 0.78%. The best path length achieved by LTSACO is always shorter than that of the other three algorithms. Moreover, in terms of average iteration time, the LTSACO algorithm demonstrates faster convergence compared to the other three, indicating its clear advantage in solving efficiency.

**Figure 14:**
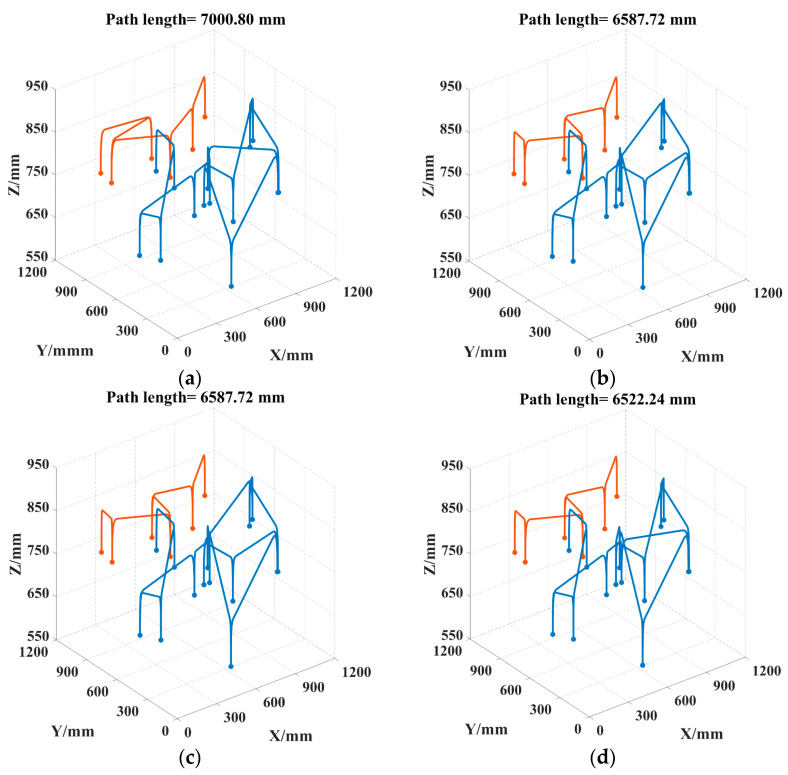
Picking routes obtained by four algorithms for 20 picking points. (**a**) ACO picking path; (**b**) DCACO picking path; (**c**) IACO picking path; (**d**) LTSACO picking path. Note: Different colors represent the motion trajectories of various picking robotic arms.

**Figure 15:**
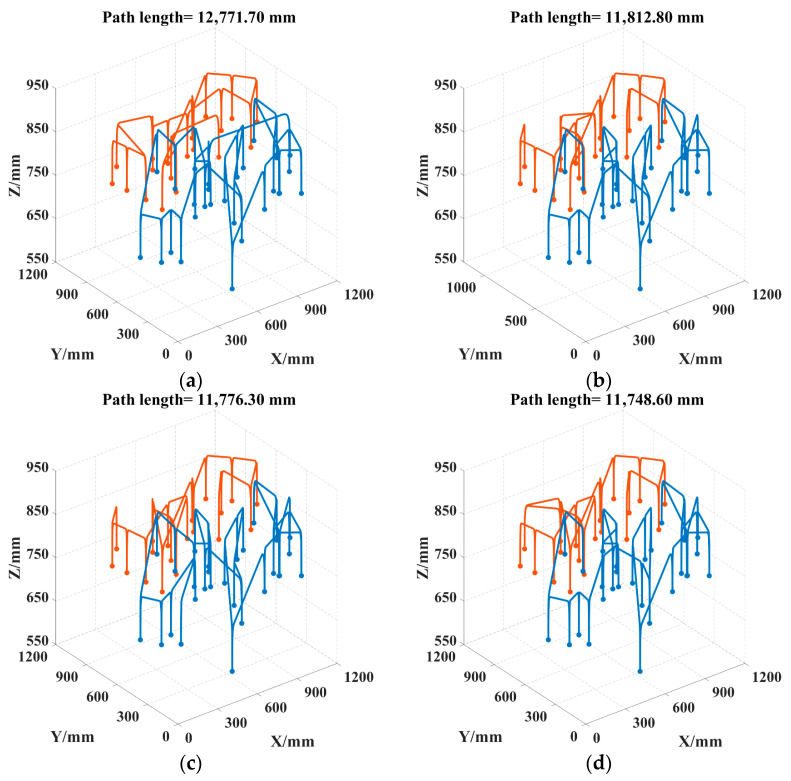
Picking routes obtained by four algorithms for 47 picking points. (**a**) ACO picking path; (**b**) DCACO picking path; (**c**) IACO picking path; (**d**) LTSACO picking path. Note: Different colors represent the motion trajectories of various picking robotic arms.

**Figure 16:**
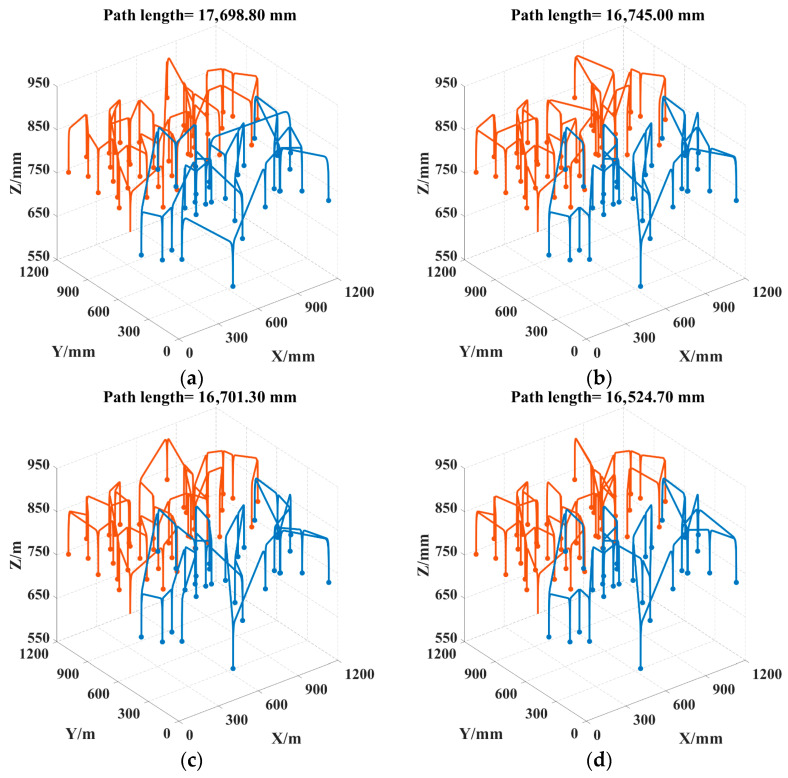
Picking routes obtained by four algorithms for 73 picking points. (**a**) ACO picking path; (**b**) DCACO picking path; (**c**) IACO picking path; (**d**) LTSACO picking path. Note: Different colors represent the motion trajectories of various picking robotic arms.

Regarding the path deviation, indicating greater volatility and lower stability. IACO shows moderate stability when the number of safflower picking points is small. In contrast, LTSACO consistently provides high-quality converged solutions across different scales, with strong robustness. The results show that LTSACO successfully overcomes the issue of getting stuck in local optima. Furthermore, regardless of the number of safflower picking points, LTSACO can plan shorter, continuous paths, making the picking process more efficient. Compared to the other three algorithms, LTSACO maintains a good convergence speed and consistently provides higher-quality solutions when the number of picking points is large. Figure 14, Figure 15 and Figure 16 show the optimal picking path visualizations for 20, 47, and 73 safflower picking points under different algorithms. The comparison of visualized paths shows that the LTSACO algorithm results in fewer path transitions.

### 4.5. Picking Experiment

**Figure 17:**
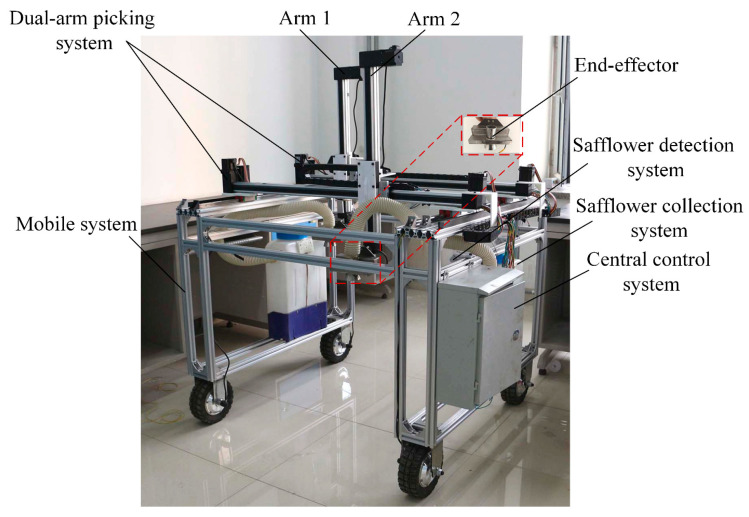
Safflower dual-arm collaborative picking experimental platform.

To verify the effectiveness of the LTSACO planning algorithm in the actual picking environment of the safflower dual-arm collaborative robot and assess the feasibility of the collaborative operation method, an experimental study is conducted using the hardware platform shown in Figure 17. The process is as follows: First, an HRnet-trained model is used to recognize the safflower images captured by the dual cameras and accurately locate the target safflowers. Next, using the calibration relationships between the world coordinate system, multi-camera coordinate systems, image plane coordinate system, and dual-arm coordinate system, the identified target coordinates are converted to the robotic arm’s tool picking coordinate system. Subsequently, MATLAB is used for path planning to generate the optimal picking path, and Python 3.9 is employed for further processing of the path planning results. G-code suitable for the STM32F407 (STMicroelectronics, Geneva, Switzerland) controller is generated and sent to the controller to drive the robotic arms to perform the picking task. The picking time and path distance are then recorded.

**Figure 18:**
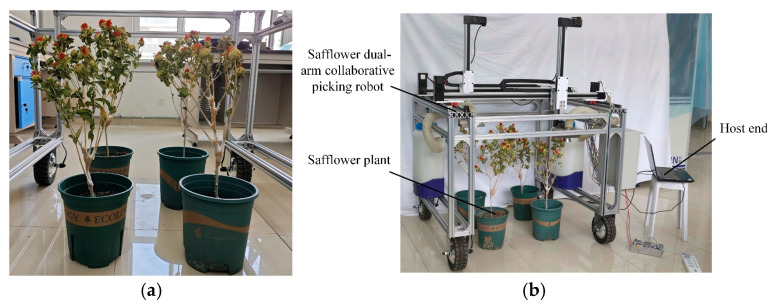
Dual-arm picking experiment. (**a**) Safflower plants; (**b**) picking experiment.

Due to the current off-season for safflower picking, artificial safflowers were used for verification, as shown in Figure 18a. The dual-arm picking test scenario is depicted in Figure 18b. In the experiment, all algorithms used the K-means algorithm to partition 46 safflower tasks, and a total of 10 repeated trials were conducted. The experimental results are presented in Table 6.

The experimental results show that the LTSACO algorithm demonstrates significant advantages in both path planning and task execution efficiency. From the perspective of path length, the experimental data indicates that its average path length is 11,577.48 mm, which is 1.20%, 0.40%, and 10.70% shorter compared to the DCACO, IACO, and ACO algorithms, respectively. This suggests that the LTSACO algorithm effectively reduces the robotic arm’s movement distance, significantly lowering energy consumption and operational time. In terms of average picking duration, the LTSACO algorithm performs in 100.5926 s, reducing the time by 2.00%, 2.60%, and 5.60% compared to the other three algorithms, further highlighting its advantage in improving task execution efficiency. Additionally, the path standard deviation of the LTSACO algorithm is only 31.64, which is significantly lower than the other algorithms, indicating the high stability and consistency of the LTSACO path planning algorithm. The primary reason for the superiority of the LTSACO path planning method over the other algorithms lies in the improved heuristic strategy, which can dynamically adjust based on runtime, effectively avoiding local optima. Furthermore, the 2-OPT local search strategy successfully eliminates path crossings, further optimizing the picking path. Combined with simulation experiments, it can be concluded that the LTSACO algorithm provides a more efficient and stable path planning solution for the safflower dual-arm collaborative picking robot, with strong robustness and reliability.

## 5. Conclusions

The dual-arm safflower picking path planning algorithm (LTSACO) proposed in this paper achieves efficient collaborative operation through multi-strategy integration. The key findings are as follows:

(1) Algorithm Innovation and Optimization Effectiveness: By improving the ant colony optimization (ACO) algorithm with a dynamic weight heuristic strategy, and combining K-means clustering for task allocation with 2-OPT local search, the algorithm reduces the path length by 7.51–11.06% and shortens the picking time by 5.60% in scenarios with 20, 47, and 73 picking points. This demonstrates the effectiveness of the balance between global search and local convergence.
(2) Trajectory Smoothing and Engineering Adaptation: A cubic Bézier curve is used to optimize the parallelogram trajectory, shortening the path length by 20.52% compared to the gate-shaped trajectory while ensuring curvature continuity, reducing arm vibrations, and meeting the motion stability requirements for high-density safflower picking.
(3) Agricultural Scenario Adaptability: The picking task is modeled as a multi-traveling salesman problem. By incorporating 3D coordinates and load balancing strategies, the algorithm addresses challenges such as plant height differences (400–950 mm) and collision-free dual-arm collaboration. In the simulation scenarios, the standard deviation is as low as 31.64, demonstrating significant robustness.

This research provides an engineering-viable optimization solution for multi-arm agricultural robots’ path planning. Future work may explore further integration of collaborative control for more robotic arms and deep integration with vision systems to enhance adaptability in complex environments.

## Figures and Tables

**Table 1:** The key parameters in the initialization algorithm.

Key Parameter	Symbol	Definition
Ant Population	*m*	Controls the diversity of the search.
Pheromone Volatilization Factor	*ρ*	Controls the rate at which pheromones decay over time.
Pheromone Factor	*α*	Adjusts the weight of pheromone concentration in path selection.
Heuristic Function Factor	*β*	Adjusts the weight of heuristic information in path selection.
Number of Clusters	*k*	Controls the expected number of clusters in the clustering algorithm.
Iteration Number	*G*	Controls the termination condition of the computation.

**Table 2:** Initialization parameters.

*m*	*ρ*	*α*	*β*	*k*	*G*
*n*	0.4	1	9	2	100

**Table 3:** Ablation experiment.

Test No.	Base Model	K-Means	Dynamic Heuristic	2-OPT	Average Path Distance/mm	Average Runtime/s
1	√	-	-	-	13,115.56	0.5416
2	√	√	-	-	11,867.66	1.2535
3	√	√	√	-	11,818.73	1.2659
4	√	√	√	√	11,780.10	1.2132

Note: In the table, the base model “Base Model” represents the original ACO model. A “√” indicates that the module has been added, while a “-” indicates that the module has not been added.

**Table 4:** Comparison of time consumption for single-arm and dual-arm.

	Type	Single-Arm Picking	Dual-Arm Picking	
Time			Area 1	Area 2
Picking Time/s		141.00	57.00	84.00
Traversal Path Time/s		117.79	48.51	68.98
Total Time/s		258.79	152.98	

**Table 5:** The results of the four algorithms under three different numbers of picking points.

Picking Points	Algorithm	Optimal Path Value/mm	Worst Path Value/mm	Average Path Value/mm	Path Standard Deviation	Average Iteration Time/s
20	ACO	7000.80	7841.02	7419.99	351.08	0.3490
	DCACO	6587.72	6983.19	6824.32	149.34	0.7410
	IACO	6587.72	6936.62	6677.55	109.51	0.5817
	LTSACO	6522.24	6861.02	6599.45	98.09	0.4024
47	ACO	12,771.70	13,318.00	12,993.35	192.62	0.4546
	DCACO	11,812.80	11,991.00	11,894.69	58.93	1.6147
	IACO	11,776.30	11,937.40	11,852.550	47.94	1.5985
	LTSACO	11,748.60	11,833.20	11,780.10	30.71	1.2132
73	ACO	17,698.80	18,900.10	18,055.80	325.53	0.5478
	DCACO	16,745.00	17,361.10	16,884.42	182.36	2.7024
	IACO	16,701.30	17,066.00	16,792.85	100.48	2.7080
	LTSACO	16,524.70	16,740.10	16,699.52	73.06	2.4973

**Table 6:** Picking test results.

Algorithm	Number of Safflowers	Single Safflower Picking Duration/s	End-Effector Travel Speed/m/s	Average Path Length/mm	Path Standard Deviation	Average Picking Duration/s
ACO	46	3	0.1	12,813.92	193.39	106.5775
DCACO	46	3	0.1	11,714.47	55.10	102.6384
IACO	46	3	0.1	11,621.15	41.24	103.3025
LTSACO	46	3	0.1	11,577.48	31.64	100.5926

## Data Availability

The data presented in this study is available on request from the corresponding author.
